# How do organisations promote the nutritional health of food manufacturing employees? A scoping review and industry survey

**DOI:** 10.1017/S1368980026102833

**Published:** 2026-06-04

**Authors:** Georgia Rogerson, Bibi Rodgers-Hunt, Louise Durrant, Hannah Theobald, Elena Philippou, Hadil Abdalla, Wendy L. Hall, Rachel Gibson

**Affiliations:** 1 Department of Nutritional Sciences, King’s College London, UK; 2 Marlow Foods Limited UK; 3 Department of Life and Health Sciences, University of Nicosia, Cyprus

**Keywords:** Workplace health, scoping review, health promotion, food manufacturing, diet behaviour

## Abstract

**Objective:**

To explore the current provision of diet and well-being interventions in UK food manufacturing businesses and identify barriers and enablers to implementation.

**Design:**

A systematic scoping review and UK industry survey were conducted. Five databases were searched (2000–2025), alongside grey literature and open-access case studies. Screening and data extraction were performed in duplicate. Intervention components were mapped to the behaviour change technique (BCT) taxonomy. An online survey (Mar–May 2025) supplemented the review by capturing current practices in UK food and beverage manufacturing. A narrative synthesis was undertaken.

**Setting:**

Food and/or beverage manufacturing sites.

**Results:**

Nineteen peer-reviewed studies (UK *n* 6, 32 %) and twelve case studies met inclusion criteria. Common BCT included ‘association’ (e.g. prompts/cues, 52 %), ‘antecedents’ (e.g. food environment restructuring, 48 %) and ‘shaping knowledge’ (e.g. nutrition education, 37 %). Most evaluations focused on dietary intake (63 %), with 47 % reporting positive changes. Key barriers were at the employee level (e.g. engagement, language and resistance to change; 42 %) and organisational level (e.g. time, staffing, funding, space; 33 %), with 25 % citing both. Survey responses (*n* 11; 82 % from large organisations) indicated interventions most often targeted ‘antecedents’ (62 %), followed by ‘shaping knowledge’ and ‘association’ (both 21 %). Budget availability and senior management support (both 45 %) were the most cited enablers in the survey.

**Conclusion:**

Evidence on nutrition and well-being interventions in UK food manufacturing is limited. Addressing implementation barriers may support wider adoption of effective behaviour change strategies.

## Introduction

Across the life course individuals will spend around 10 % of their time at work^(1)^. While employment promotes physical and mental health and supports social and economic stability^(2)^, the workplace is a key contributor to determinants of health. A UK economic and public health priority is to increase healthy working life span^(3)^. The workplace is a key environment for public health nutritional interventions, with research demonstrating that promotion of nutritional health is of benefit to businesses and employees^(4)^. In 2023, The Nutrition Society Workplace Diet and Health Special Interest Group Round Table Meeting identified a research priority to understand how to engage hard-to-reach employee groups (e.g. low paid, male, shift workers, ethnic minorities)^(5)^ with nutrition and well-being interventions. Food and beverage is the largest manufacturing industry in the UK^(6)^ employing ∼400 000 staff^(7)^ and is essential to sustaining UK food supply. Many of these employees have atypical working hours (e.g. outside 7 a.m. to 7 p.m., weekends, long hours and zero-hour contracts) and tend to be in low-paid occupational groups. Strong evidence supports the association between poor cardiometabolic health with shift work^(8)^ and long working hours^(9)^, potentially mediated by poor dietary choices^(10)^. An extensive systematic review showed that workplace interventions with a nutrition component can improve dietary intake and cardiometabolic health but highlighted a need to understand how to tailor workplace programmes across different socio-economic contexts^(11)^. This may be of particular importance to UK food manufacturing staff, as shift workers have been shown to have limited engagement with workplace health programmes,^(12)^ and the wider manufacturing sector has been shown to have low investment in staff well-being programmes^(13)^.

Interventions to promote improved workforce health need to be of a complex design (e.g. targeting multiple levels – from individuals to society and/or comprising of multiple components). The UK guidance for developing complex interventions recommends that interventions should target key identified behaviours^(14)^. The Behaviour Change Wheel is an established toolbox recommended to inform UK public health intervention development, from selection of target behaviours to mapping behaviours to interventions that are under pinned by behaviour changes techniques^(15)^. An important development stage is to understand the types of behavioural interventions that are currently implemented or have been previously evaluated as well as to also understand implementation barriers and enablers.

Despite the size and economic importance of the UK food and beverage manufacturing sector, little is known about how employers support the nutritional health of their workforce, particularly those in low-paid or shift-based roles. Occupational challenges that are likely to contribute to nutritional health and well-being include working in highly controlled environments (e.g. protective clothing, temperature-controlled), manual and repetitive tasks and sensory exposure to food aromas. Evidence on the types of interventions implemented and the practical barriers to delivery remains limited. To address this gap, a scoping review and industry survey were conducted to: (i) characterise the nature and content of workplace diet and well-being interventions in food manufacturing settings; (ii) map intervention components to the Behaviour Change Techniques (BCTs) Taxonomy and (iii) identify perceived barriers and enablers to implementation. Findings will inform the development of more effective, context-specific interventions to promote food and beverage manufacturing employees’ health in this hard-to-reach UK workforce.

## Methods

This project combined a systematic scoping literature review (Open Science Framework registration https://osf.io/3pxku/overview) and a UK food and beverage manufacturing industry survey to characterise the key elements of programmes that have been tested or implemented. A scoping review methodology was selected as the aim was to characterise the types of research conducted within this field and to identify knowledge gaps^(16)^.

### Scoping review

A systematic scoping review was conducted following JBI guidelines^(17)^. The search strategy aimed to identify published and unpublished sources of evidence. An electronic database search was conducted in the following databases: MEDLINE, EMBASE, APA PsycINFO and Web of Science. The search strategy was constructed using the Population, Concept and Context framework. The search strategy, including all identified keywords and index terms, was adapted for each database and/or information source. Reference lists of systematic reviews and included articles were hand screened for additional studies. Unpublished (‘grey literature’) sources were searched and included trial registers (US National Institute of Health (clinicaltrials.gov), ISRCTN and WHO (ISCTRP registry)), PhD theses (UK repositories), preprints, organisational websites and key conference papers were searched for relevant studies, for full details of the search see Supplementary material – search strategy.

Peer reviewed studies and grey literature publications were included if they reported exposure to a workplace nutrition, health and well-being intervention set in a high-income country (defined by The World Bank Classification), among food or beverage manufacturing sector employees. The aim of the overall project is to develop strategies for UK organisations. However, as the pilot search identified a limited number (*n* 6), consensus agreement of the research team was to include studies in any high-income country to capture a wide number of examples that may have applicability to the UK. The food and beverage manufacturing sector was defined in consultation with the UK Food and Drink Federation as *‘companies that are primarily engaged in producing and/or processing and packaging final goods for human consumption from raw and/or processed food materials’.* Manufacturing employees were defined as having a job role related to production operations in food or beverage manufacturing, covering roles such as production operatives, lab technicians to engineers. Where there was uncertainty over definitions, experts from the project steering group were consulted. There was no exclusion criteria applied to participant characteristics such as age, health status or ethnicity. Searches were limited to English language publications from January 2000 January 2025. Low- and middle-income settings were excluded as were those conducted in agriculture, food retail, catering and food service. Studies with no diet or nutrition component to the intervention were also excluded.

### Study selection

All identified citations were collated and uploaded into Covidence literature review software (https://www.covidence.org/) and duplicates removed. Following a pilot, titles and abstracts were screened by two independent reviewers (GR, HA) against the inclusion and exclusion criteria. Any disagreements between reviewers were resolved by a third reviewer (RG). Potentially relevant citations were retrieved in full, and full text assessed for inclusion by two independent reviewers (RG, HA). Reasons for exclusion at full text stage were recorded and presented in a Preferred Reporting Items for Systematic Reviews and Meta-analyses extension for Scoping Reviews^(18)^. Any disagreements arising between reviewers were resolved by a third reviewer (RG). See reporting checklist in Supplementary material.

### Industry case studies

An extensive internet search was performed to identify publicly available case studies where an employee nutrition, health or well-being intervention had been implemented within the food or beverage manufacturing industry. An industry case study was defined by the research team as a publicly available published description or evaluation of a health and well-being programme in a food manufacturing company. All case studies identified were collated into a Microsoft Excel spreadsheet and screened by two independent reviewers (GR, HA) against the above inclusion criteria. Any disagreements were resolved by a third reviewer (RG), Supplementary material.

### Data extraction and Behaviour Change Technique coding

Data were extracted in duplicate (GR and HA) using a data extraction template developed through consensus of the research team members (RG, BRH, WH, EP, LD and HT). The extraction tool was piloted by GR and HA prior to data extraction. The following information was extracted: Study design, industry setting, number of employees, participant characteristics, method of programme delivery (e.g. in person and by whom), type of nutrition intervention(s) employed, any non-nutrition intervention components, intervention duration, the method and measures of evaluation used, available information on levels of employee engagement, reported barriers or enablers to implementation, funding source. The characteristics of the nutrition programme or intervention elements describe in each study were extracted and coded (e.g. provision of healthier canteen options was coded as physical food environment change) and then mapped to one of sixteen groups of BCTs listed in The BCTs Taxonomy (BCTTv1)^(19)^. Each BCT is defined as *‘an active component of an intervention designed to change behaviour’* ^(20)^. For example, a change in the physical food environment (e.g. addition of healthy menu items in a canteen) was mapped to Group 12 ‘*Antecedents*’ – a stimulus that triggers a behaviour. Some intervention characteristics were mapped to two BCT groups, e.g. free fruit delivery was mapped to Group 7 ‘*Associations*’ – a stimulus with the purpose of prompting or cueing the behaviour and Group 12 *‘Antecedents’* as the provision of fruit also add a healthy food into the environment. Coding and mapping were conducted by two independent reviewers (GR, HA). Any disagreements arising in coding were resolved by a third reviewer (RG).

### Results reporting and synthesis

Characteristics of the studies included are described using descriptive statistics. A narrative synthesis was conducted to identity behavioural techniques applied across all data sources. The reported barriers and enablers to intervention implementation were collated and mapped to the social ecological model based on the level they referred to: macro-wider society, community level, organisational level and employee level.

### Industry survey

The industry survey was a self-administered online open survey (Supplementary material) and reported in-line with the Checklist for Reporting Results of Internet E-Surveys (CHERRIES) guidelines^(21)^, Supplementary material CHERRIES checklist. Informed consent was obtained from all subjects at the start of the survey.

The web-based survey was built using Qualtrics, version 2025 (https://www.qualtrics.com) and divided into the following seven sections, reflecting the same domains of interest as the scoping review: organisational setting, nutrition, health and well-being provision, worksite food provision, nutrition support, staff participation, evaluation and feedback and, barriers and enablers to implementation. Before survey distribution, the survey was piloted among five people (researchers, dietitians and steering group members). Feedback was gained on the survey’s layout, clarity, content, ease and logic. Adaptive questioning was applied throughout to reduce complexity of questions, and forced responses were set to prevent incomplete responses. A non-response option, such as ‘prefer not to say’ or ‘I’m not sure’, was provided for all questions, no back button was provided. The order of questions was not randomised. The final survey contained thirty-three questions over thirteen pages, with a combination of multiple-choice questions and free text. The survey collected responses from individuals involved in staff nutrition, health and well-being across the UK food and beverage manufacturing industry. The survey ran between 27 March and 5 May 2025.

The survey was emailed to a list of 650 industry contacts via gatekeepers, and it was also advertised on social media (LinkedIn) through The Nutrition Society, British Dietetic Association and Nutritionists in Industry (professional networking and continuous professional development group for nutritionists working in industry). Respondent eligibility was self-reported; working directly or indirectly in nutritional health and well-being of UK food manufacturing employees. This was a convenience sample with no a priori sample size because of the exploratory and descriptive nature of the study. Participants were advised of the survey length, data storage procedures, lead investigator and the purpose of the survey on the landing page. Informed consent was obtained at this point. Participants were advised that survey completion was voluntary, and they could stop at any point during the survey; however, once submitted, participants were advised that they would be unable to withdraw their responses. As an incentive to participate, respondents were invited to enter a prize draw to win one of two £50 shopping vouchers following completion of the survey. To participate in the prize draw, participants were directed to a separate Qualtrics link for them to enter their email address and consent to the prize draw. It was not possible to link the email addresses to the participant responses, and all email addresses were deleted after the prizes had been claimed. Before analysis, all incomplete responses were excluded. Narrative synthesis was performed and the results triangulated with scoping review findings.

## Results

### Scoping review

**Figure 1.**
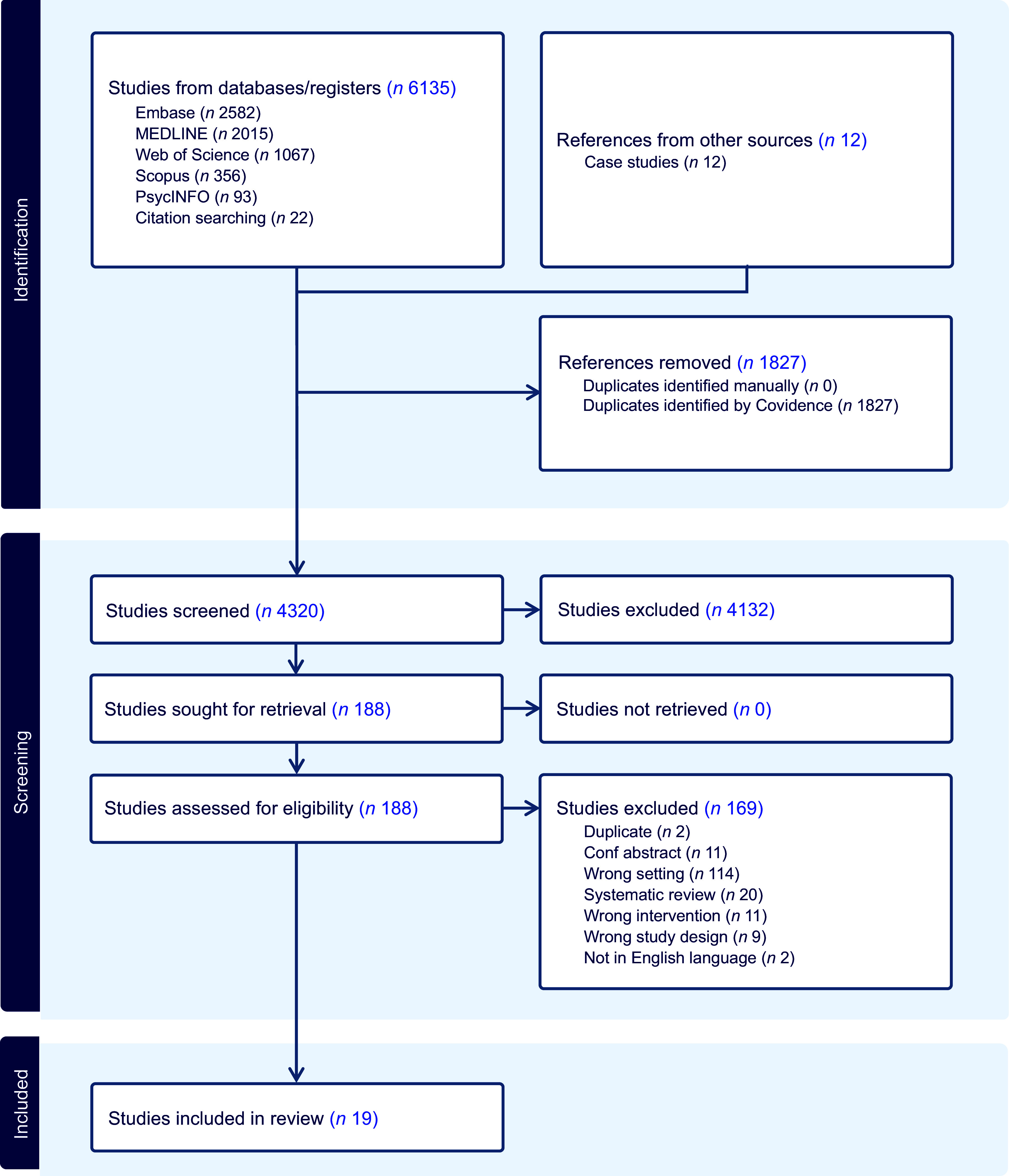
Figure 1 long description. PRISMA-SCR flow diagram.

Of the 6147 studies screened, nineteen published studies met the inclusion criteria and are included in this review, as shown in Figure 1. Six studies were conducted in a UK setting^(22–27)^, six in Europe,^(28–33)^, three in the USA^(34–36)^, two in Asia^(37,38)^ and two in Australasia^(39,40)^. Except in three studies^(28,35,39)^, the company name was not reported. Five studies reported the baseline health status of employees, with overweight/obesity prevalence ranging 30 %–100 %, the latter in a study focused on male workers living with obesity^(37)^ from characteristics of published studies are shown in Table 1.

**Table 1.** Characteristics of studies included in the systematic scoping review

Ref	Sector and worksites	Occupation	Country	Company name	Participant numbers	Health status
Alexopoulos 2014^(33)^	Food processing (honey)	Blue collar workers, food scientists, technologists, supervisors	Greece	NR	20	39 % BMI >= 25 kg/m^2^
Backman 2011^(34)^	9 worksites, food manufacturing (2 sites) *, apparel manufacturing (7 sites)	low wage workers	USA	NR	559	NR
Caloyeras 2014^(35)^	Food and beverage processing	NR	USA	PepsiCo	44 408	NR
Cook 2001^(39)^	2 manufacturing worksites, one meat processing, one electronics	Blue collar hourly employees	New Zealand	Fisher and Paykel refrigeration and Auckland Meat Processing company	253	40 % living with obesity; 30 % with elevated blood pressure
Holdsworth 2000^(23)^	4 worksites, one is a crisp manufacturer head office	11 % manual workers	UK	NR	577	31 % living with overweight or obesity
Holdsworth 2004^(22)^	4 worksites, one is a crisp manufacturer head office	11 % manual workers	UK	NR	577	31 % living with overweight or obesity
Hollands 2018^(24)^	9 worksites, all members of IGD	Unskilled or semi-skilled workers	UK	NR	1018/7173 participants at manufacturing sites	NR
Iriyama 2013^(37)^	5 worksites, including 3 food manufacturing sites	Intervention arm 39 % manufacturing or sales, 61 % administration. Control arm 52 % manufacturing or sales, 48 % administration	Japan	NR	79	Study population is male workers with obesity
Pechey 2019^(25)^	6 worksites (1 depot, 3 offices and 2 manufacturing sites). Sites recruited through IGD	Managerial, administrative and professional occupations, supervisory, clerical and junior managerial, semi-skilled and unskilled manual occupations	UK	NR	1083/5200 participants at manufacturing sites	NR
Roy 2020^(40)^	22 companies within the NZFGC	NR	New Zealand	NR	NR	NR
Sawada 2015^(38)^	1 food manufacturing worksite	NR	Japan	NR	900	Changes in BMI reported
Sorensen 2003^(36)^	15 worksites manufacturing: Adhesives, food, technology, jewelry, motor controls, paper products, newspaper, abrasives, automobile parts and metal fabrication	Management, workers	USA	NR	NR	NR
Van Holland 2017^(32)^	5 worksites in meat-packing industry	Managers, human resources, occupational health services	The Netherlands	NR	986	NR
Van Holland 2018^(31)^	5 worksites in meat-packing industry	NR	The Netherlands	NR	986	NR
Van Scheppingen 2014^(30)^	Dairy company	Managers, human resources and employees	The Netherlands	NR	324	NR
Vasiljevic 2018^(27)^	6 worksites, all members of IGD	Higher managerial, intermediate managerial, supervisory of clerical/junior managerial, skilled manual worker, semi or unskilled manual workers	UK	NR	5208	NR
Vasiljevic 2019^(26)^	3 worksites, all members of IGD	Higher managerial, intermediate managerial, supervisory, clerical, junior managerial skilled, manual workers, semi or unskilled workers	UK	NR	2947	NR
Velema 2018^(29)^	30 worksites: chemical, automotive, electronic, power engineering, food, finance and insurance, industries to government institutions, facility and entertainment industries	79·5 % white collar workers	The Netherlands	NR	NR	NR
Vitale 2018^(28)^	Food manufacturing company	50 % plant workers, 50 % office workers	Italy	Barilla S.p.A.	738	NR

* Food manufacturing sites were allocated to control condition. IGD, The Institute of Grocery Distribution; NZFGC, New Zealand Food and Grocery Council, NR, not reported.

### Published studies – intervention components and outcome measures

**Figure 2.**
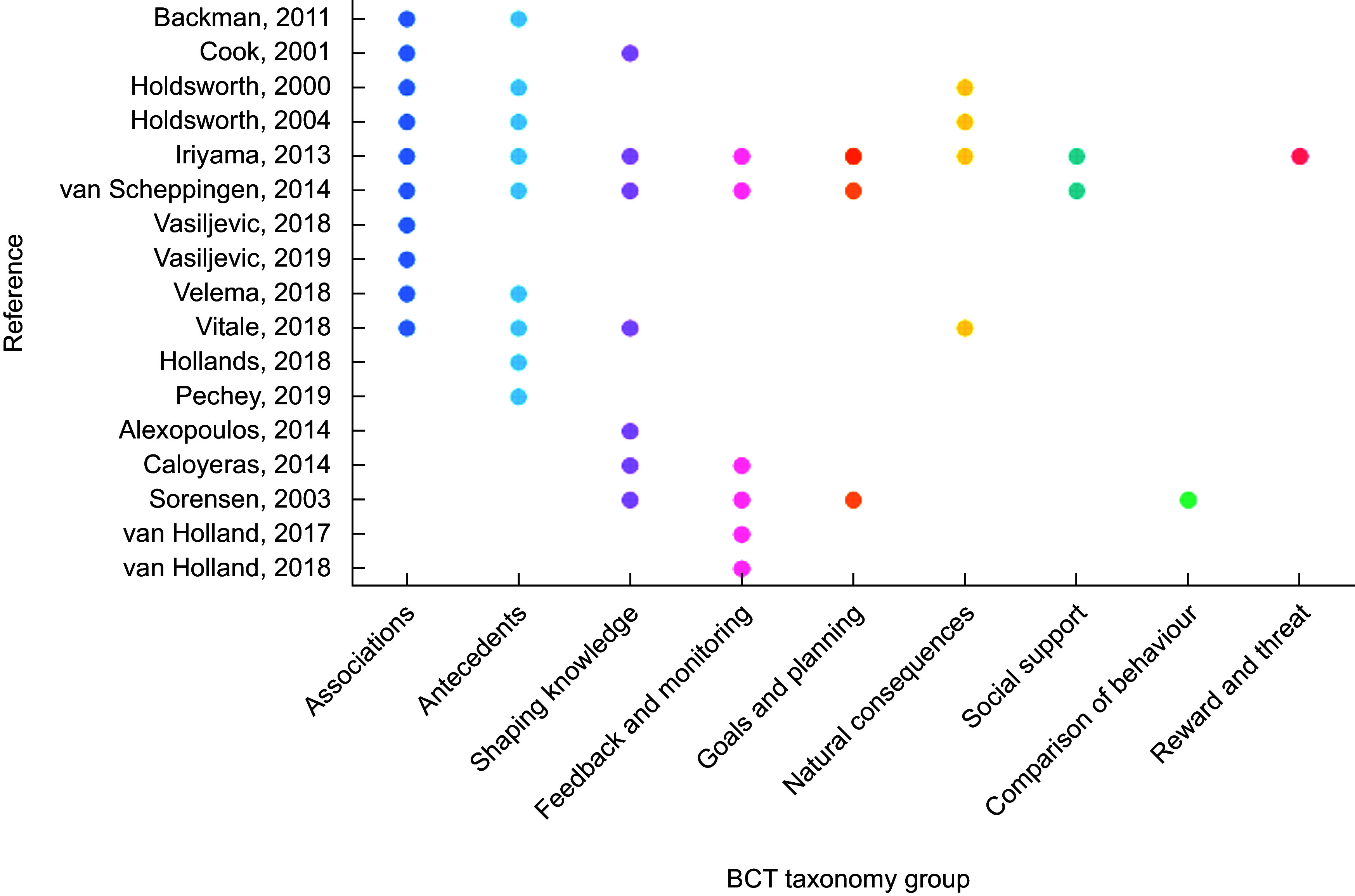
Mapping behaviour change techniques (BCT) to identified elements of nutrition interventions in published studies. Two studies had insufficient detail to enable mapping.

From the sixteen groups of BCT in the taxonomy^(19)^, elements of the nutritional interventions identified were mapped to nine of them, in ten studies interventions were mapped to more than one BCT group – the most common combination being ‘*Associations’* and ‘*Antecedents’* in seven studies, Figure 2, supplementary Table S1. The most frequently mapped was Group 7 ‘*Associations’* with ten studies (53 %) having intervention elements mapped^(22,22,26–30,34,37,39)^, intervention elements mainly included the provision of cue and prompts through provision of fruit^(30,34)^, canteen labels^(26,27)^ and material highlighting healthier choices^(22,23,28,29,37,39)^. Nine studies (47 %) had intervention elements mapped to Group 12 ‘*Antecedents’* ^(22–25,28–30,34,37)^, these were mainly around restructuring the physical or social environment – for example, providing free fruit and altering canteen menu availability to focus on healthy options. Seven studies (37 %) were mapped to Group 4 ‘*Shaping knowledge’* ^(28,30,33,35–37,39)^ intervention elements included education and workshop sessions^(28,30,33,35–37,39)^ and provision of educational materials^(22,23,28,33,37,39)^. Six studies (32 %) were mapped to Group 2 ‘*Feedback and monitoring’* ^(30–32,35–37)^ with elements mainly including health assessments that included feedback to staff. Four studies (21 %) mapped to Group 5 *‘Natural consequences’* ^(22,23,28,37)^, three studies (16 %) to Group 1 *‘Goals and planning’* ^(30,36,37)^, two studies (11 %) from group 3 ‘*Social support‘* ^(30,37)^, one study (5 %) from Group 6 ‘*Comparison of behaviour’* ^(36)^ and one study (5 %) from Group 10 ‘*Reward and threat*’^(37)^. Non-nutrition intervention components were also reported, these included alcohol awareness, counselling, food hygiene advice, fitness or physical activities, smoking cessation and provision of no smoking areas. Characteristics of interventions implemented, mapped BCT and findings are shown in Table 2.

**Table 2.** Nutrition, health and well-being interventions, reported barrier, enablers and summary results Table 2 long description.

Study	Nutrition intervention	Non-nutrition interventions	Outcome(s) measured	Barriers – authors reported	Enablers – authors reported	Findings
Alexopoulos 2014^(33)^	Group education, printed educational materials	NR	Nutrition knowledge (score) Dietary habits (score)	The good general health of participants might have diminished scope for improvements	NR	Nutrition knowledge +ve Dietary habits 0
Backman 2011^(34)^	Free fruit deliveries	NR	Fruit and vegetable consumption, fruit and vegetable purchasing, self-efficacy towards eating 2 servings fruit and vegetables per day, job satisfaction, perceived health	Low employee response rate at control sites	NR	Fruit and vegetable consumption, fruit purchasing, vegetable purchasing, self-efficacy toward eating 2 servings of fruit per day +ve Job satisfaction, Perceived health 0
Caloyeras 2014^(35)^	Health screening, individual education (referral to LM or DM pathways)	Stress management, smoking cessation, fitness	Healthcare costs, absenteeism, ROI	NR	NR	Healthcare costs DM pathway +ve, LM pathway 0 Absenteeism DM pathway 0, LM pathway +ve ROI returned USD 3·78 (DM) and USD 0·48 (LM)
Cook 2001^(39)^	Group education, printed educational materials, point of choice messages	Non-communicable disease risk, alcohol awareness, benefits of physical activity	% consuming 2–3 servings fruit per day, % consuming 2–3 servings veg per day, % consuming breakfast, % consuming >6 drinks per session, fat intake (score), nutrition knowledge score, BMI, weight, WC, blood pressure.	Low participation rate (42 %)	Key workers were consulted during planning and the stages of change model guided development and delivery	Fruit intake, breakfast intake, heavy drinking, BMI, weight, WC 0 Vegetable intake, fat intake, nutrition knowledge, SBP +ve
Holdsworth 2000^(23)^	Healthy food options, food hygiene, provision of non-smoking area, printed educational health materials	Good food hygiene, provision of non-smoking area	Nutrition knowledge (of dietary guidelines)	NR	NR	Nutrition knowledge 0
Holdsworth 2004^(22)^	Healthy food options, food hygiene, provision of non-smoking area, printed educational health materials	Good food hygiene, provision of non-smoking area	Diet quality	NR	NR	Fruit, fried foods, sweet puddings, lower fat milk intakes +ve Biscuits, confectionery, sugary drinks, processed meat, cheese, crisps, beans and pulses, veg, chicken and fish, starchy food, red meat and low energy drinks 0
Hollands 2018^(24)^	Reducing portion sizes	NR	Total energy (kcal) purchased per day from target categories, Total energy (kcal) purchased per day from all categories	Employee resistance to portion size reductions, but general approval of proportional pricing strategies.	The worksite cafeterias are quasi-commercial (not operated solely for profit), should management wish to support changes to the cafeterias, commercial concerns may take less precedence	Energy purchased from target and all categories 0
Iriyama 2013^(37)^	Individual education session, group education sessions, printed educational materials, point of choice messages, healthy menu	Stress management, advice on social drinking, one exercise advice session	Body weight (kg), BMI (kg/m^2^), waist circumference (WC), BP (mm/Hg), TAG (mg/dL), cholesterol (mg/dL), blood glucose (fasting plasma glucose and HbA1c), aspartate aminotransferase (IU/dL), alanine aminotransferase (IU/dL)	NR	NR	Body weight, BMI, alanine aminotransferase +ve WC, BP, cholesterol, blood glucose, aspartate aminotransferase 0 Triglycerides –ve
Pechey 2019^(25)^	Increasing healthier options available (measured by kcal)	NR	Total energy (kcal) purchased per day from intervention categories, total energy (kcal) purchased per day from all categories, Total revenue per day from all categories	No indication of potential cost to organisation for implementation. Small-scale change, long period of implementation. Customer resistance to change	NR	Energy purchased from intervention categories, Energy purchased from all categories +ve Total revenue 0
Roy 2020^(40)^	Cross-sectional survey, focus group discussions	NR	WEA survey measured health promotion in the workplace	Time and lack of structure in the running or implementation of various programmes within the policy, physical space constraints	NR	WEA score indicated an ‘average’ commitment to health-promotion. Larger food companies scored higher than smaller companies
Sawada 2015 ^(38)^	Lifestyle/diet survey, health screening	NR	Association of vegetable consumption with risk of weight gain	NR	NR	Risk of weight gain +ve (sig lower) in highest *v*. lowest quartile of vegetable consumption
Sorensen 2003 ^(36)^	Consultation on catering and cafeteria policies, self-help activities, contests, demonstrations, goal setting, group discussions	Consultation on exposure to hazardous substances used in work processes, tobacco control policies	Smoking quit rates (all workers, hourly workers, salaried workers), fruit and vegetable intake (servings)	Reliance of self-report, representation of a self-selected group	NR	Smoking quit rate +ve in hourly workers, 0 in salaried workers Fruit and vegetable intake 0
Van Holland 2017 ^(32)^	Biometric heath screening, individual advice on follow-up	Functional capacity evaluation, counselling sessions	Process evaluation	Resistance from work floor supervisors who had to adjust planning. The programme took more time than anticipated	Government support, Dutch law requires large employers to offer suitable occupational healthcare to employers	The average reach of the program was 53 %. Participants were satisfied with the program
Van Holland 2018 ^(31)^	Biometric heath screening, individual advice on follow-up	Functional capacity evaluation, counselling sessions	Absenteeism (individuals with >9 d per year), workability (WAI score), productivity (QQ score), health status (EUROQoL score), vitality (RAND-36 score), psychosocial workload (COPSOQ II score), ROI	Program not available to all employees	NR	Health status, vitality, psychosocial workload 0 Absenteeism, workability, productivity –ve ROI -3·9 EUR returned for 1 EUR spent
Van Scheppingen 2014 ^(30)^	Group activities, workshop, provision of fruit/healthy snacks	Physical activities	Social capital, openness towards health, smoking, healthy dietary habits, sustainable employability, autonomous motivation towards a healthy lifestyle, physical activity, alcohol use, relaxation, health and vitality at work	Low engagement rate	Managers and employees enabled to make their own specified next step to health promotion, appropriate to their needs	Linear regression reported. Bonding social capital, openness towards health, smoking, healthy dietary habits and sustainable employability +ve Autonomous motivation towards a healthy lifestyle, physical activity, alcohol use, relaxation or health and vitality at work 0
Vasiljevic 2018 ^(27)^	Calorie labelling	NR	Total energy (kcal) purchased	NR	NR	Total energy (kcal) 0
Vasiljevic 2019 ^(26)^	Increased prominence of calorie labelling	NR	Total energy (kcal) purchased	NR	NR	Total energy (kcal) 0
Velema 2018 ^(29)^	Healthier meal options, product placement, meal deals, pricing strategies, healthy products named first on menu.	NR	Warm snacks, fruit, prepackaged snacks, healthier sandwiches, healthier salads, healthier cheese, healthier meat purchased	NR	NR	Linear regression reported. Fruit, healthier sandwiches, healthier cheese +ve Warm snacks, prepackaged snacks, healthier salads, healthier meat 0
Vitale 2018 ^(28)^	Nutritional education for food preparation staff, menu modification, newsletter, printed educational materials	NR	Adherence to recommended intakes for protein, fat, saturated fat, cholesterol, carbohydrate, sugars and fibre. Wholegrain bread, wholegrain pasta, refined bread, refined pasta, legumes, vegetables and fruit, white meat, red meat, fish, eggs cheese and cold cuts, soft drink intakes.	NR	Program is feasible and effective	Adherence to recommendations for saturated fat, cholesterol, sugars, fibre +ve Adherence to recommendations for protein, fat, carbohydrate 0 Wholegrain bread and pasta, legumes, white meat and fish intake increased. Refined bread and pasta, red and processed meat, eggs and cheese decreased Vegetable and fruit, soft drink intake 0

BCT, behaviour change technique taxonomy (17); LM, lifestyle management; DM, disease management; SBP, systolic blood pressure; ROI, return on investment; WC, waist circumference; WEA, workplace environment audit; NR, not reported. Legend: +ve Significant positive change, –ve significant negative change, 0 no significant change.

In total, ninety-five outcomes were reported in published studies. These were categorised into dietary outcomes (*n* 63), health outcomes (*n* 20) and organisational outcomes (*n* 12). These were wide ranging, including the following: Adherence to dietary recommendations, breakfast intake, dietary quality, energy purchased, fat intake, fruit and vegetable intake, intakes of healthy and unhealthy food items, nutrition knowledge, absenteeism, healthcare costs, job satisfaction, productivity, vitality and workability, blood glucose, blood pressure, BMI, body weight, cholesterol, perceived health, TAG and waist circumference. Of the sixty-three dietary outcomes, thirty-three (52 %) reported a positive change^(22,25,28–30,33,35,39)^, thirty (48 %) reported no significant difference^(22–24,26–29,33,36,39)^. Of the 20 health outcomes, seven (35 %) reported a positive change^(30,36,37,39)^, one (5 %) reported a negative change^(37)^ and, 12 (60 %) reported no significant difference^(30,37,39)^. Of the twelve organisational outcomes, five (42 %) reported a positive change^(30,35)^, three (25 %) reported a negative change^(31)^ and four (33 %) reported no significant difference^(30,31)^.

### Case studies – intervention components

**Figure 3.**
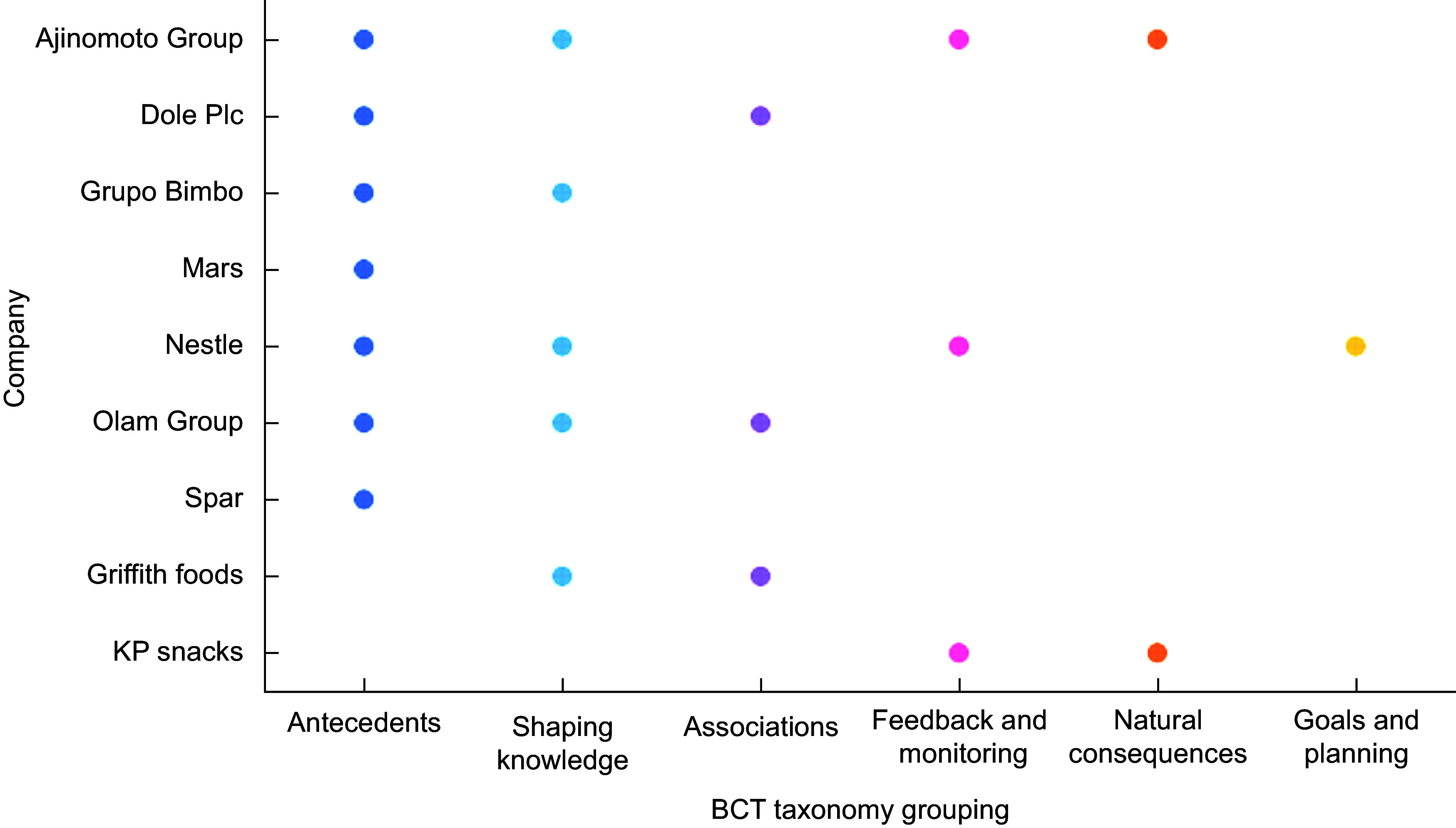
Mapping behaviour change techniques (BCT) to identified elements of nutrition interventions in case studies. Three studies had insufficient detail to enable mapping.

Twelve case studies met the inclusion criteria^(41–48)^. These were predominantly conducted across company sites globally, five were focused primarily on UK and Republic of Ireland locations. Characteristics of case studies are shown in Table 3. A range of nutrition interventions were reported, and elements were mapped to seven of the sixteen BCT groups, as shown in Figure 3. Seven companies were identified as using BCT elements from Group 12 *‘Antecedents*’, for example restructuring the physical food environment through healthy canteen options and vending. Five companies had elements mapped to Group 4 ‘*Shaping knowledge*’, including nutrition education sessions (one-one, webinars and workshops), provision of educational materials. Three companies had elements mapped to Group 2 *‘Feedback and monitoring’ e.g.* health screenings, three companies from Group 7 *‘Associations’*, two companies from Group 5 ‘*Natural consequences’* one company from Group 1 ‘*Goals and planning’*. Non-nutrition intervention components were reported in all case studies, these included addiction prevention, breastfeeding support, breathing exercises, cycle to work schemes, ergonomic support, family and community events, financial and legal advice, fitness or physical activities, food hygiene advice, mental health support, physiotherapy, sleep advice, smoking cessation and provision of a smoke-free environment. None of the case studies reported the outcomes measured as part any evaluation of the programmes.

**Table 3.** Characteristics of case studies identified

Company name	Country	Intervention	BCT(s) used	Non-nutrition components	Barriers noted	Enablers noted
Ajinomoto Group ^(41)^	Case study focuses on Japan, Spain, Brazil, Thailand	Healthy cafeteria menu, nutrition education sessions, seminars, individual health consultations, digital resources, app	2·6 Biofeedback, 4·1 Instruction on how to perform a behaviour, 5·1 Information about health consequences, 12·1 Restructuring the physical environment	Annual health checks, Yoga classes, Exercise advice, Sleep advice	NR	NR
Dole Plc ^(42)^	USA	Healthy menu with subsidised healthy options, healthy vending, twice daily free fruit/veg breaks, access to onsite dietitian, biweekly newsletters, weekly farmers market	7·1 Prompts/cues, 12·1 Restructuring the physical environment	Access to physical activities	Expectation that some employees will be unhappy with the changes ‘burly men will not want to be forced to eat tofu’	Senior management support, funding
Ferrero Group ^(43)^	Global	Nutrition education	NR	Awareness days, Fitness challenges, Ergonomic support, Preventative healthcare, Mental health support, Financial and legal advice, Family and community events	NR	NR
Griffith Foods ^(44)^	Global	Nutrition education, nutrition labelling in canteen	4·1 Instruction on how to perform a behaviour, 7·1 Prompts/cues	NR	Language barriers, balancing complexity of messages across diverse groups of workers	Use of internal nutrition leads enables tailored messages based on employee feedback
Grupo Bimbo ^(45)^	Global	Healthy cafeteria menu, nutrition education, workshops/talks, nutrition health checks	4·1 Instruction on how to perform a behaviour, 12·1 Restructuring the physical environment	Physical activity campaigns, Mental health support, Disease prevention campaigns, Preventative health check-ups, Addiction prevention, Ergonomic assessment, Breast-feeding support	Global challenges in adapting menus to local tastes, translating materials, and ensuring alignment with associate needs.	Use of WNA resources offers diverse perspectives and best practices. Tailoring initiatives to global contexts. WNA masterclass for HR staff to implement effective strategies internally
KP Snacks ^(46)^	Case study focuses on UK	Provision of health kiosks, self-checking of BP and health statistics, interactive platform including recipes	2·6 Biofeedback, 5·1 Information about health consequences	Workouts, Financial education, Mental health support	NR	NR
Mars ^(46)^	Global, case study focuses on UK	Healthy food and drink offering, discounted pricing on healthier options	12·1 Restructuring the physical environment	Mental health resources, awareness days. Gym memberships, Cycle 2 work schemes. Pilates, Yoga, Meditation classes, Walk events, Ergonomically designed communal eating spaces, Physiotherapy	NR	NR
Mondelez International Inc. ^(46)^	UK	Advice ‘Help employees look after their diet’, health stations for employees to create personalised reports	NR	Health and fitness classes with discounted memberships. Menopause and cancer support initiatives. Mental health, financial well-being support. Team forums to foster community and belonging	NR	NR
Nestle ^(46)^	Case study focuses on UK and Ireland	Health check with biofeedback and health coaching, healthy eating education, minimum nutritional standards across worksite canteens	1·4 Action planning, 2·6 Biofeedback, 4·1 Instruction on how to perform a behaviour, 12·1 Restructuring the physical environment	Mental health support, Hybrid and pet friendly working, Financial education	Turnover of occupational health staff	Investment (in staff)
Olam Group ^(47)^	Unclear if LIC and MIC only	Healthy food and drink offering, nutrition education, health checks	4·1 Instruction on how to perform a behaviour, 7·1 Prompts/cues, 12·1 Restructuring the physical environment	Breast-feeding support	NR	NR
PepsiCo ^(46)^	Likely Global, case study focuses on UK and Ireland	Online educational resources, webinars, awareness days	NR	Sleep, exercise and money management advice, Mental health ambassadors at some sites	NR	NR
Spar ^(48)^	Spain	Healthy food and drink offering	12·1 Restructuring the physical environment	Physiotherapy, Information on accident prevention, Promotion of sports facilities, Stress techniques, Smoke-free environment, Breathing exercises, Managers to consider breast-feeding needs, Promotion of healthy work relationships	NR	Changes to physiotherapists schedule based on employee requests

BCT, behaviour change technique taxonomy; LIC, low-income country; MIC, middle-income country; NR, not reported; WNA, Workforce Nutrition Alliance.

Fewer BCT were attributed to interventions reported in case studies compared with published studies (seven *v*. nine BCT coded), with higher frequency of physical environment modifications coded from case studies (30 % case studies *v*. 19 % published papers).

### Industry survey

Despite a potential high survey reach, the response rate was low. In total, twenty-eight respondents started the survey, of these, 12 (43 %) completed all questions. One participant response was excluded in the final analysis due to ineligibility (not food and beverage manufacturing setting). One participant completed the survey in a paper copy format on request. Nine respondents reported working with the food manufacturing sector, one with beverage manufacturing and one did not state. Respondents included dietitians and nutritionists, human resources, occupational health, health and well-being managers, company directors, communications and business development officers. The majority (91 %) of respondents worked with large (>250 employees) sized companies, employing staff on permanent, fixed term and zero hours contracts. Five (45 %) respondents reported having no staff canteen providing hot meals at manufacturing sites, and three (27 %) made no food service provision for out of hours (weekend or night) working. Characteristics of survey respondents and settings are shown in Table 4. A range of nutrition interventions were reported, including provision of healthy cafeteria food (*n* 8, 73 %), educational materials (*n* 7, 64 %), water/drinks machines (*n* 7, 64 %), nutrition labelling (*n* 4, 36 %), free fruit deliveries (*n* 3, 27 %), discounts on healthier options (*n* 3, 27 %) and cooking demonstrations (*n* 2, 18 %). Non-nutrition intervention components were also reported, these included mental health support (*n* 8, 73 %), health awareness advice such as alcohol or smoking cessation (*n* 7, 64 %), eye tests (*n* 7, 64 %), ergonomic assessments (*n* 6, 55 %), fitness/physical activities (*n* 6, 55 %), vaccination clinics (*n* 5, 45 %), sleep advice (*n* 2, 18 %), dental plans (*n* 1, 9 %), remote GP access (*n* 1, 9 %) and physiotherapy (*n* 1, 9 %). Private health insurance provision was reported at five companies, although if this was offered to all staff or only those above a certain level was unclear.

**Table 4.** Characteristics of stakeholders who responded to the UK food and drink manufacturing staff health and well-fbeing implementation survey

	Number of responses	%
**Industry sector**		
Food	9	82
Beverage	1	9
Not reported	1	9
**Respondent job role**		
Dietitian/nutritionist	2	18
Human resources	2	18
Occupational health	2	18
Other^1^	4	
Prefer not to say	1	9
**Manufacturing headcount**		
<49	1	9
50–249	0	0
250–999	5	45
>1000	4	36
Unsure	1	9
**Staff canteen**		
No	5	45
Yes, some sites	3	27
Yes, all sites	2	18
Unsure	1	9
**Out of hours food service**		
No	3	27
Yes, some sites	4	36
Yes, all sites	3	27
Unsure	1	9
**Food heat/storage facilities**		
No	1	9
Yes, all sites	9	82
Unsure	1	9

1 Health & Wellbeing Manager, Company Director, Communications Manager, Business Development Officer.

### Identified behaviour change elements mapped to intervention function and Theoretical Domains Framework

**Figure 4.**
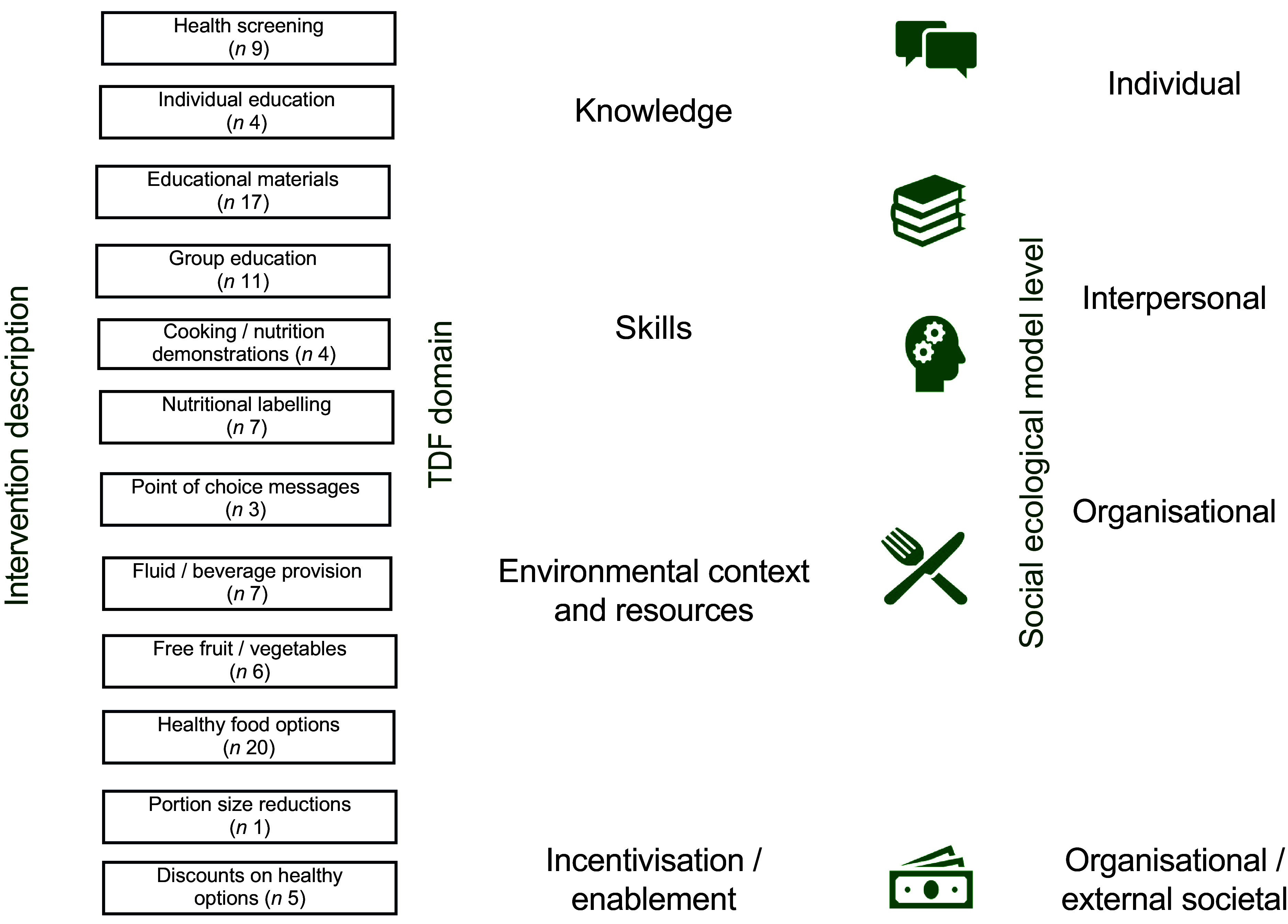
Figure 4 long description. Summary of identified nutritional behavioural change interventions across published studies, case studies and survey respondents mapped to the theoretical domains framework and social ecological model. TDF, theoretical domains framework.

Most intervention elements identified from all data sources were focused at the individual level in the ‘knowledge’ domain – for example, health screening and individual education, as shown in Figure 4. Limited interventions were identified as being in the ‘skills’ domain; demonstrations along with group education are targeted at the ‘interpersonal’ level – as these activities are likely to occur with groups of colleagues. The second most frequent domain identified was ‘environmental context and resources’ – modifications in the food environment, for example – provision of water, fruit, vegetables and healthy choices. The only example of incentivisation was the provision of discounted healthy foods.

### Barriers and enablers across published studies, case studies and survey respondents

**Figure 5.**
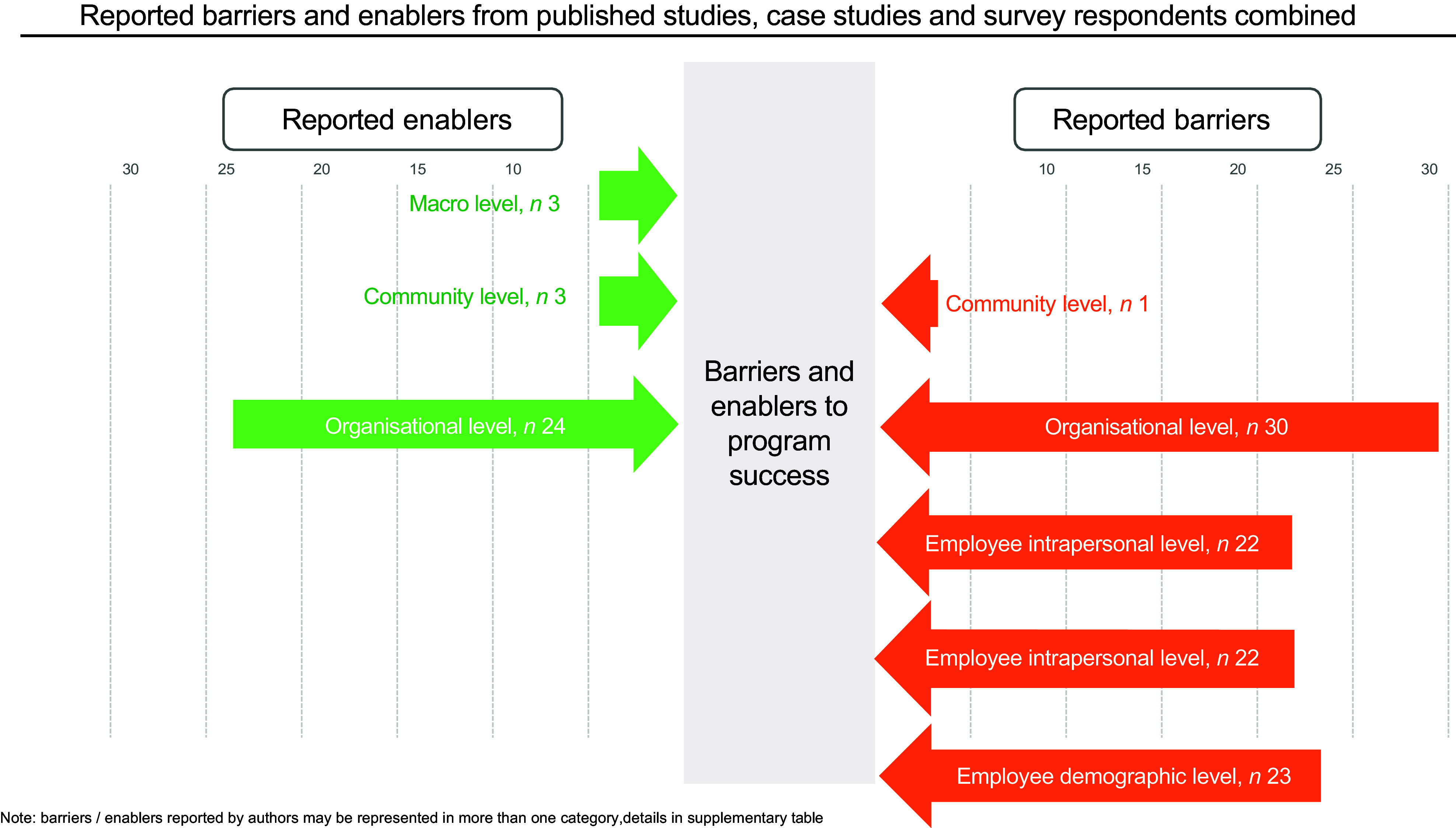
Reported barrier and enablers from published studies, case studies and survey respondents combined. Examples included at each level. Macro level, i.e. government incentives/legislation, community level, i.e. global challenges adapting to local tastes, links to local health provisions, organisational level, i.e. production demands, lack of senior sponsorship, employee interpersonal level, i.e. language barrier, employee resistance to change, employee intrapersonal level, i.e. food preferences, low engagement rate, employee demographic level, i.e. good health limiting scope for improvement, language barrier.

Ten published studies and four cased studies reported barriers to implementation or limitations to success. Common barriers to implementation of nutrition and well-being interventions were reported consistently across published studies, organisational case studies and survey responses. These were mapped to employee, organisational and macro-level factors drivers, as shown in Figure 5. At the employee level, low engagement, resistance to change, language barriers and food preferences were frequently cited. Published articles noted: employee resistance to change^(24,25)^ and low employee engagement rate^(30,34,39)^ – quantified in two studies as 28 % and 42 % uptake by eligible staff. The reasons noted in case studies included employee resistance to change (*n* 1), language barriers (*n* 1), global challenges adapting menus and messaging to local tastes (*n* 1) and turnover of occupational health staff (*n* 1). From interview responses, eight (73 %) respondents noted lack of staff interest and 27 % stated language barriers, Table 5. Organisational barriers included limited resources (time, space, staff or budget), unsupportive working patterns, lack of supervisor or senior management buy-in and practical constraints related to shift work or food service provision. From studies included in the scoping review, organisational-level barriers included that the programme was not available to all employees^(31)^, lack of supervisor support^(32)^, too lengthy/time consuming^(32,40)^, reliance on self-reported information (36), lack of implementation structure and physical space constraints^(40)^. Production demands were noted by five (45 %) respondents to the survey, and employees being too busy (27 %), work environment/facilities not suitable (27 %), lack of training/expertise to deliver initiatives (9 %) and lack of senior-level sponsorship and supervisor support were also noted.

**Table 5.** Intervention elements, barriers and enablers reported by stakeholders involved in UK workplace interventions in the food and beverage manufacturing sector

	Number of responses	%
**Nutrition interventions reported mapped to BCT taxonomy**		
Group 12 Antecedents:		
Healthy food options	8	73
Water coolers/drinks machines	7	64
Discounts on healthy options	3	27
Group 7 Associations:		
Nutrition labelling	4	36
Free fruit	3	27
Group 4 Shaping knowledge:		
Educational materials	7	64
Group 6 Comparison of behaviour:		
Cooking demonstrations	2	18
**Non nutrition interventions reported**		
Mental health support	8	73
Health awareness advice	7	64
Eye tests	7	64
Ergonomic assessments	6	55
Physical activities	6	55
Vaccination clinics	5	45
Sleep advice	2	18
Dental plans	1	9
GP access	1	9
Physiotherapy	1	9
**Barriers to implementation reported**		
Lack of staff interest	8	73
Staff working patterns	8	73
Production demands	5	45
Food preferences	4	36
Language barrier	3	27
Staff too busy	3	27
Environment/facilities not suitable	3	27
Lack of training/expertise	1	9
Lack of senior sponsorship or supervisor support	1	9
**Enablers that would support implementation**		
Making budget available for initiatives	5	45
Senior sponsorship or supervisor support	5	45
Allowing employees time to access initiatives	4	36
Government incentives	1	9

BCT, behaviour change technique.

Several enablers were also identified, including strong leadership support, employee involvement in programme design, flexible delivery models and internal capacity (e.g. in-house nutrition leads). Enabling factors stated in published studies included consultation with workers during planning stage^(39)^, stages of change model guided programme development and delivery^(39)^, not-for-profit cafeteria allowed commercial concerns to take less precedence^(24)^, managers and employees enabled to make their own appropriate next steps toward health^(30)^ and government legislation requiring employers to offer suitable occupational healthcare^(32)^. Five case studies reported strengths or enablers to implementation including senior management support and funding (*n* 1), internal nutrition provider allows tailored messaging based on employee feedback (*n* 2), use of external resources allows for diverse perspectives and best practices (*n* 1), tailoring initiatives to global contexts (*n* 1), investment (*n* 1) and flexibility in providers schedule based on employee requests (*n* 1). From survey responses, budget available for initiatives was noted by five (45 %) as well as senior-level sponsorship and supervisor support (45 %) and allowing time for employees to access initiatives during work (36 %). Survey respondents also highlighted the potential role of external incentives such as government grants or community partnerships to improve uptake and sustainability of workplace health initiatives. Community partners and/or external support, government guidelines, supervisor training, and senior-level sponsorship were suggested as possible resources that would help to improve employee nutrition, health and well-being in the food manufacturing setting.

## Discussion

Occupational health behaviours and longer‑term health outcomes are shaped by the interaction between workplace environments and wider social and structural determinants. In the UK food and beverage manufacturing sector, a workforce disproportionately employed in low‑paid roles, with a high representation of migrant workers^(49)^ and exposure to shift work and physically demanding labour, may be particularly vulnerable to diet‑related health inequalities. Diet is a key modifiable behaviour, with diets that are low in fibre, fruit and vegetables and high in salt, sugar and saturated fat contributing significantly to morbidity and mortality in the UK population^(50)^. This scoping review demonstrates a limited and weakly evaluated UK evidence base for workplace nutrition interventions in manufacturing settings, with existing initiatives predominantly focused on environmental modification and education, and recurrently constrained by low employee engagement, organisational barriers and a lack of whole‑systems integration.

Health interventions in workplaces have the potential to engage with hard-to-reach population groups including ethnic minority groups and male employees. These population groups are overrepresented in manufacturing job roles that require atypical working schedules and are at the lower end of salary scales. The COVID pandemic and Brexit have made staff recruitment and retention a challenge across the UK food and beverage manufacturing sector^(4)^. These roles are vital to the resilience of the UK food system. This scoping project aimed to understand current practice and the barriers and enablers to supporting the nutritional health of UK food and beverage manufacturing workers in the workplace. The results point to a lack of evaluation in UK settings with most interventions utilising knowledge enhancement and environmental cues; however, a lack of employee engagement and resistance to change were identified as the main barriers to successful implementation. Previous research has proposed reasons for poor engagement levels that may be the result of demographic, language, cultural barriers and lack of availability within working hours^(51)^. Effective behaviour change interventions should be tailored to the population and underpinned by behaviour change theory^(20)^. This project found that most interventions used BCT that target modification of the food environment – primarily the addition of healthier food options, often coupled with prompts and cues, that aim to ‘nudge’ individuals towards a specific behaviour. A combination of ‘nudge’ strategies in workplace food provision has been shown to improve whole grain purchasing, but not across other food categories^(52)^. However, studies tend to be short in duration; therefore, limited data exist on the sustainable dietary changes that are needed for improved health outcomes, for example, within a school setting health outcomes are anticipated after 5 years of a nutritional programme^(53)^. Within a manufacturing context, the timing of food availability is key – as many employees in the sector work outside of standard working hours. Survey results suggested that not all sites have out of hours food service provision that would limit the reach of any workplace canteen nudge strategies. Shaping knowledge – providing information through nutritional health education and promotion sessions – was a common element of interventions but rarely used in isolation. Previous studies have suggested that multi-component interventions are more likely to be effective than those addressing only one element^(14)^.

Behaviours around food choice and nutrition at work are influenced by several domains – interventions should target the behaviours identified as being key determinants. The identified barriers to implementation and success were similar across those reported by published articles, case studies and survey – with most citing individual staff-level barriers, e.g. employee resistance to change and lack of engagement, language barriers and individual food preferences. Future research needs to understand from an employee perspective how to develop interventions to address these barriers. Wider organisational barriers included higher management support and practical considerations such as space, capability and production demands. In terms of enablers, published sources included higher stakeholder support and recourses. Survey respondents stated that wider support was needed such as government incentives and engagement with community health partners. These observations point to the need for a whole systems approach to workforce nutritional health, integrating interventions at multiple levels across individual staff, team, organisation and policy. Rather than isolated initiatives, a whole systems approach aligns leadership, culture, environment and employee support systems to improve workforce health and organisational performance.

The purpose of a scoping review is not to determine the strength of the evidence, but to understand what has been published in this topic and the elements of interventions that have been implemented. The scoping review identified limitations of the current evidence base. There is a lack of studies conducted in a UK setting, this is important to address as the UK health system structure is different to many other countries, for example, in America where most organisations are mandated to provide health care and incentivised for health promotion. The population characteristics were poorly reported particularly ethnicity, job roles and contract type (e.g. permanent or agency staff). The setting of the intervention was not always clearly reported to determine the legibility. Other fields that were unclearly reported were the intervention lead and if other components were delivered alongside the nutrition intervention, e.g. physical activity. In addition, there was limited information regarding the health status of food manufacturing staff, this may be likely to lack of information available (e.g. would not be collected routinely in some organisations). Although the case studies provided information around interventions and programmes, they lacked outcome measures. There are several likely reasons for this including confidentiality and commercial sensitivity – this should be further explored to enable effective knowledge exchange.

### Strengths and limitations

A key strength of the scoping review is the number of databases and grey literature searched and the addition of an industry survey. The scoping review followed the JBI methodology with independent reviewing and data extraction. An additional strength is the involvement of the steering committee comprising of expert stakeholders independent of the research, this ensured that the terms of reference were appropriate throughout the scoping project. The poor reporting of industry sector may mean that food and beverage manufacturing-based studies were missed due to not specifying the sector. The mapping of intervention elements to BCT is based on an element of subjectivity, and to strengthen the scoping review, the BCT were mapped independently, and disagreements resolved by the research team. The BCT mapping is based on the information reported in the published papers and case studies; some interventions may have used elements that were not reported. The industry survey was limited by the low response rate; there are several reasons that could have impacted this including sector pressures around time to complete, bias around not wanting to complete the survey unless positive interventions to report and lack of identifiable personnel who see themselves as responsible with nutritional well-being for this occupational group. This latter point may be a reason why most responses were from large organisations that are more likely to have a designated member of staff in this role. Based on our scoping review, a full systematic review is not indicated at this time due to the variability in sectors, interventions and measurements reported.

### Implications and next steps

Future research in this area would benefit from fully reporting of intervention details, for example, application of Template for Intervention Description and Replication checklist,^(54)^ this would support potential synthesis of data to fully understand intervention characteristics Table 6. While studies conducted outside of the UK can be useful, given the size of the UK food and manufacturing workforce, the paucity of studies limits UK-specific knowledge.

**Table 6.** Supporting workplace nutrition and well-being in UK food and beverage manufacturing employees – recommendations for research and practice

Area	Recommendation
Design and implementation	Involve a wide range of employees in co-development. Consider inclusivity across job role especially those with high workload. Adoption of a whole systems approach.
Evaluation and reporting	Follow reporting guidelines to enable replication. Report occupational and demographic information. Include qualitative evaluation. Disseminate all evaluations – via case studies, published papers or webinars to share knowledge.
Policy	Government initiatives/support for businesses. For example, wider promotion of workplace health and well-being initiatives that are free at the point of access (WHISPA) ^(55)^.

The next stage of this project is to conduct a qualitative study with employees employed in the UK food manufacturing sector to understand their perceptions and view around workplace nutrition health and well-being from future research we can then consider the barriers and enablers from an employee perspective and how behavioural change techniques can be developed to support health of UK food and manufacturing employees. Given the number of studies identified in this scoping review, a full systematic review is indicated to determine the effectiveness of interventions.

## Conclusion

Despite food and drink manufacturing being the largest UK manufacturing sector, the scoping review and industry survey highlight a notable lack of evaluated nutrition and well-being interventions. Those that exist predominantly focus on modifying the food environment, providing education and prompts/cues, with a limited use of a broader range of BCTs. Barriers to implementation, particularly low employee engagement, practical constraints of shift work and resource limitations, are consistently reported across evidence sources, meriting further exploration. To promote better workforce health in this sector, future research should prioritise co-development of interventions with a tailored, broader range of BCTs that are feasible and acceptable. Programmes should be evaluated across a range of metrics including levels of engagement across the whole workforce. This in turn may support investment and budget availability for sustained well-being programmes.

## Supporting information

10.1017/S1368980026102833.sm001 Rogerson et al. supplementary material 1 Rogerson et al. supplementary material

10.1017/S1368980026102833.sm002 Rogerson et al. supplementary material 2 Rogerson et al. supplementary material

## Supplementary material

For supplementary material accompanying this paper visit https://doi.org/10.1017/S1368980026102833

## Figure 1. Long description

The flowchart illustrates the process of identifying, screening, and including studies in a review. It begins with the identification phase, where studies are sourced from databases/ registers and other references. Studies from databases/ registers include Embase (n = 2582), MEDLINE (n = 2015), Web of Science (n = 1067), Scopus (n = 356), PsycINFO (n = 93), and citation searching (n = 22), totaling 6135 studies. References from other sources include case studies, totaling 12 studies. In the next step, 1827 references are removed due to duplicates identified by Covidence. The screening phase involves 4320 studies, of which 4132 are excluded. 188 studies are sought for retrieval, with none not retrieved. These 188 studies are assessed for eligibility, resulting in 169 studies being excluded for various reasons such as being duplicates, conference abstracts, wrong setting, systematic reviews, wrong intervention, wrong study design, and not being in English. Finally, 19 studies are included in the review.

Navigate back to Figure 1..

## Table 2. Long description

A table with 16 rows and 7 columns. The columns are labeled Study, Group, BCT, Intervention, Outcome, Barriers, and Enablers. The table lists various studies and their corresponding BCT groups, interventions, outcomes, barriers, and enablers. Each row provides detailed information about the specific study, the BCT group to which the intervention elements were mapped, the type of intervention, the outcome of the intervention, and the barriers and enablers reported. The table captures the diversity of interventions and their effects across different studies.

Navigate back to Table 2..

## Figure 4. Long description

A diagram mapping nutritional behavioral change interventions to the theoretical domains framework and social ecological model. The diagram is divided into two main sections: Intervention description and TDF domain on the left, and Social ecological model level on the right. The Intervention description section lists various interventions such as Health screening, Individual education, Educational materials, Group education, Cooking/nutrition demonstrations, Nutritional labelling, Point of choice messages, Fluid/beverage provision, Free fruit/vegetables, Healthy food options, Portion size reductions, and Discounts on healthy options. These interventions are categorized under Knowledge, Skills, Environmental context and resources, and Incentivisation/enablement. The Social ecological model level section categorizes these interventions into Individual, Interpersonal, Organizational, and Organizational/external societal levels. Each category is represented by different icons: speech bubbles for Individual, stacked books for Interpersonal, a head with gears for Organizational, a fork and knife for Organizational, and money for Organizational/external societal.

Navigate back to Figure 4..
