# Supplementary material for: How do organisations promote the nutritional health of food manufacturing employees? A scoping review and industry survey
Source: Public Health Nutr. 2026 Jun 4;29(1):e133. doi: 10.1017/S1368980026102833 (PMC13434577; doi:10.1017/S1368980026102833)
Supplement: Supplementary file 1 [file S1368980026102833sup001.docx]

**How do organisations promote the nutritional health of food manufacturing employees? A scoping review and industry survey.**

**Database search strategy:**

| **Category** | **Inclusion criteria** | **Search terms** |
| --- | --- | --- |
| **Population** | - Adult employees >16 | **Concept 1 Keywords: combine with OR** workforce*; workplace*; worksite* worker* ((work* or employment* or labour or labor) adj2 (place* or site* or setting* or environment* or force*)) employee*; employer*; staff; occupation*; personnel **Concept 1 Subject headings:** **Ovid Medline MeSH terms:** workforce **Ovid Embase Emtree terms:** workforce **Ovid PsychInfo MeSH terms:** workforce tbc **WOS NO SUBJECT HEADINGS** |
| **Concept** | - Nutrition intervention | **Concept 2 Keywords: combine with OR**  ((nutrition* or diet* or food or eating or health* or lifestyle or wellbeing or well being or cooking) adj2 (polic* or program* or intervention* or campaign* or promotion* or education or habit* or preference* or intake* or behaviour* or behavior* or practice* or choice* or option* or status or therap* or quality or change or knowledge))  ((weight or obesity or diabetes or cardiovascular) adj2 (management or loss or maintenance or reduction or prevention))  ((calor* or fat* or salt* or fiber or fibre or sugar* or fruit* or vegetable* or carbohydrate* or energy or fluid or protein or beverage*) adj2 (intake* or consume or consumes or consumption or consumed or diet*)).  **Concept 2 Subject headings:**  **Ovid Medline MeSH terms:** diet therapy OR nutrition therapy OR health promotion  **Ovid Embase Emtree terms:** nutrition education OR nutrition policy OR health promotion  **Ovid PsychINFO MeSH terms:** diet therapy OR health promotion tbc  **WOS NO SUBJECT HEADINGS** |
| **Context** | - (UK and EUR) Food manufacturing setting | **Concept 3 Keywords: combine with OR** ((food or drink or beverage) adj1 (production or process* or industry or handling or manufactur* or factory or plant or enterprise or facility or site)) **Ovid Medline MeSH terms:** food industry OR food handling **Ovid Embase Emtree terms:** Food industry OR food handling **Ovid PsychINFO MeSH terms:** food industry OR food handling tbc **WOS NO SUBJECT HEADINGS** |
| **1 AND 2 AND 3** | | |
| **Filter English language, published 2000 to date, manually screen for UK and EUR settings** | | |

EMBASE

1.workforce*.tw.2.workplace*.tw.3.worksite*.tw.4.worker*.tw.5.((work* or employment*or labour or labor) adj2 (place* or site* or setting* or environment* or force*)).tw.6.employee*.tw.7.employer*.tw.8.staff.tw.9.occupation*.tw.10.personnel.tw.11.exp workforce/12.((nutrition* or diet* or food or eating or health* or lifestyle or wellbeing or well being or cooking) adj2 (polic* or program* or intervention* or campaign* or promotion* or education or habit* or preference* or intake* or behaviour* or behavior* or practice* or choice* or option* or status or therap* or quality or change or knowledge)).tw.13.((weight or obesity or diabetes or cardiovascular) adj2 (management or loss or maintenance or reduction or prevention)).tw.14.((calor* or fat* or salt* or fiber or fibre or sugar* or fruit* or vegetable* or carbohydrate* or energy or fluid or protein or beverage*) adj2 (intake* or consume or consumes or consumption or consumed or diet*)).tw.15.exp nutrition education/16.exp nutrition policy/17.exp health promotion/18.((food or drink or beverage) adj1 (production or process* or industry or handling or manufactur* or factory or plant or enterprise or facility or site)).tw.19.exp food industry/20.exp food handling/21.1 or 2 or 3 or 4 or 5 or 6 or 7 or 8 or 9 or 10 or 1122.12 or 13 or 14 or 15 or 16 or 1723.18 or 19 or 2024.21 and 22 and 2325.limit 24 to english language

MEDLINE

| 1. | workforce*.tw. |
| --- | --- |
| 2. | workplace*.tw. |
| 3. | worksite*.tw. |
| 4. | worker*.tw. |
| 5. | ((work* or employment*or labour or labor) adj2 (place* or site* or setting* or environment* or force*)).tw. |
| 6. | employee*.tw. |
| 7. | employer*.tw. |
| 8. | staff.tw. |
| 9. | occupation*.tw. |
| 10. | personnel.tw. |
| 11. | exp workforce/ |
| 12. | ((nutrition* or diet* or food or eating or health* or lifestyle or wellbeing or well being or cooking) adj2 (polic* or program* or intervention* or campaign* or promotion* or education or habit* or preference* or intake* or behaviour* or behavior* or practice* or choice* or option* or status or therap* or quality or change or knowledge)).tw. |
| 13. | ((weight or obesity or diabetes or cardiovascular) adj2 (management or loss or maintenance or reduction or prevention)).tw. |
| 14. | ((calor* or fat* or salt* or fiber or fibre or sugar* or fruit* or vegetable* or carbohydrate* or energy or fluid or protein or beverage*) adj2 (intake* or consume or consumes or consumption or consumed or diet*)).tw. |
| 15. | exp diet therapy/ |
| 16. | exp nutrition policy/ |
| 17. | exp health promotion/ |
| 18. | ((food or drink or beverage) adj1 (production or process* or industry or handling or manufactur* or factory or plant or enterprise or facility or site)).tw. |
| 19. | exp food industry/ |
| 20. | exp food handling/ |
| 21. | 1 or 2 or 3 or 4 or 5 or 6 or 7 or 8 or 9 or 10 or 11 |
| 22. | 12 or 13 or 14 or 15 or 16 or 17 |
| 23. | 18 or 19 or 20 |
| 24. | 21 and 22 and 23 |
| 25. | limit 24 to english language |

APA PSYCINFO

| 1. | workforce*.tw. |
| --- | --- |
| 2. | workplace*.tw. |
| 3. | worksite*.tw. |
| 4. | worker*.tw. |
| 5. | ((work* or employment*or labour or labor) adj2 (place* or site* or setting* or environment* or force*)).tw. |
| 6. | employee*.tw. |
| 7. | employer*.tw. |
| 8. | staff.tw. |
| 9. | occupation*.tw. |
| 10. | personnel.tw. |
| 11. | exp workforce/ |
| 12. | ((nutrition* or diet* or food or eating or health* or lifestyle or wellbeing or well being or cooking) adj2 (polic* or program* or intervention* or campaign* or promotion* or education or habit* or preference* or intake* or behaviour* or behavior* or practice* or choice* or option* or status or therap* or quality or change or knowledge)).tw. |
| 13. | ((weight or obesity or diabetes or cardiovascular) adj2 (management or loss or maintenance or reduction or prevention)).tw. |
| 14. | ((calor* or fat* or salt* or fiber or fibre or sugar* or fruit* or vegetable* or carbohydrate* or energy or fluid or protein or beverage*) adj2 (intake* or consume or consumes or consumption or consumed or diet*)).tw. |
| 15. | exp nutrition education/ |
| 16. | exp nutrition policy/ |
| 17. | exp health promotion/ |
| 18. | ((food or drink or beverage) adj1 (production or process* or industry or handling or manufactur* or factory or plant or enterprise or facility or site)).tw. |
| 19. | exp food industry/ |
| 20. | exp food handling/ |
| 21. | 1 or 2 or 3 or 4 or 5 or 6 or 7 or 8 or 9 or 10 or 11 |
| 22. | 12 or 13 or 14 or 15 or 16 or 17 |
| 23. | 18 or 19 or 20 |
| 24. | 21 and 22 and 23 |
| 25. | limit 24 to english language |

**Grey search terms**

**(workplace or worksite or worker or staff or employee) and (nutrition or diet or lifestyle or intervention or program or intake) and (food or drink or beverage or manufacturing or processing or factory or plant)**

| **Trials registers** | Clinical trials.gov | no relevant results found |  |
| --- | --- | --- | --- |
|  | ISRCTN | no relevant results found |  |
|  | ICTRP | no relevant results found |  |
| **PhD theses** | OATD | no relevant results |  |
|  | Proquest | no relevant results |  |
|  | WorldCat | no relevant results found |  |
| **Preprints** | MedRxiv | no relevant results found |  |
| **Websites/conferences/case studies** | Society of occupational medicine | no relevant info | [Home \| The Society of Occupational Medicine](https://www.som.org.uk/) |
|  | What works wellbeing | No relevant info | https://whatworkswellbeing.org/ |
|  | Wellbeing at work conference Feb 2025 | No relevant info | [CONFERENCE AGENDA - Wellbeing At Work Conference](https://events.cipd.co.uk/wellbeing-at-work-conference/conference-agenda) |
|  | Wellbeing at work summit 2024 | No relevant info | [Wellbeing at Work Summit UK 2024 in London & Manchester](https://wellbeingatwork.world/summit/wellbeing-at-work-summit-uk-2025/) |
|  | Worktech | No relevant info | [Event Site Archive - WORKTECH - Unwired Ventures Ltd](https://www.worktechevents.com/events/) |
|  | Workforce wellbeing | No relevant info | [Workforce Wellbeing](https://informaconnect.com/workforce-wellbeing/) |
|  | IGPP | No relevant info | [Public Policy Research Papers and Resources \| IGPP](https://igpp.org.uk/research) |
|  | Nut Soc | No relevant info | [The home of evidence-based nutritional science \| The Nutrition Society](https://www.nutritionsociety.org/) |
|  | BDA (Work Ready) | No relevant info | [Home - British Dietetic Association (BDA)](https://www.bda.uk.com/) |
|  | FDF case studies | case studies: Pepsico, Mars, Mondelez, KP, Nestle | [Workforce and employment case studies \| The Food & Drink Federation](https://www.fdf.org.uk/fdf/resources/case-studies/workforce-and-employment-case-studies/?SearchTerm=&Tag=&SortOrder=&PageSize=8&Show=) |
|  | Workforce Nutrition | Case study: Ajinomoto foods | https://workforcenutrition.org/nutrition-without-compromise-the-ajinomoto-group-case-study/ |
|  |  | Case study: Griffith foods | https://workforcenutrition.org/griffith-foods-commitment-to-workforce-nutrition/ |
|  |  | Case study: Grupo Bimbo | https://workforcenutrition.org/grupo-bimbo-bakes-a-healthier-workforce-a-recipe-for-global-well-being/ |
|  |  | Case study: Olam (OFI) | https://workforcenutrition.org/employee-health-and-well-being-olam-food-ingredients-ambition-to-workforce-nutrition-programmes/ |
|  |  | Case study: Ferrero | https://www.theconsumergoodsforum.com/case-study/wellbeing-matters-global-awareness-strategy-a-ferrero-case-study/ |
|  |  | Case study: Spar | https://www.theconsumergoodsforum.com/case-study/spar-te-cuida-health-programme/ |
|  |  | Case study: Mars | https://www.mars.com/sustainability-plan/nourishing-wellbeing/be-well-workplace |
|  | Food Manufacture | No relevant info | https://www.foodmanufacture.co.uk |
|  | HSE | No relevant info | https://www.hse.gov.uk/index.htm |
|  | Nutrition Connect | Case study: Dole | https://nutritionconnect.org/sites/default/files/uploads/resources/2019-04/Food%20at%20Work%20-%20ILO.pdf |
|  | GAIN | No relevant info | https://www.gainhealth.org |
|  | RAND | No relevant info | https://www.rand.org |
|  | Nutritionists in industry | No relevant info | <https://nii.org.uk/> |
|  | Dietitians in industry | No relevant info | twitter |
|  | Royal Society for Public Health | No relevant info | https://www.rsph.org.uk |
|  | YourWellspace | Case study: Douwe Egberts | https://yourwellspace.com/corporate-wellbeing-in-the-food-production-industry/ |
