# Supplementary material for: How do organisations promote the nutritional health of food manufacturing employees? A scoping review and industry survey
Source: Public Health Nutr. 2026 Jun 4;29(1):e133. doi: 10.1017/S1368980026102833 (PMC13434577; doi:10.1017/S1368980026102833)
Supplement: Supplementary file 2 [file S1368980026102833sup002.docx]

**Table S1 Intervention and outcome reporting details for studies included in the scoping review**

| **Study** | **BCT(s) targeted** |
| --- | --- |
| Alexopoulos, 2014 | 4.1 Instruction on how to perform a behaviour |
| Backman, 2011 | 7.1 Prompts/cues, 12.5 Adding objects to the environment |
| Caloyeras, 2014 | 2.6 Biofeedback, 4.1 Instruction on how to perform a behaviour |
| Cook, 2001 | 4.1 Instruction on how to perform a behaviour, 7.1 Prompts/cues |
| Holdsworth, 2000 | 5.1 Information about health consequences, 7.1 Prompts and cues, 12.1 Restructuring the physical environment |
| Holdsworth, 2004 | 5.1 Information about health consequences, 7.1 Prompts and cues, 12.1 Restructuring the physical environment |
| Hollands, 2018 | 12.1 Restructuring the physical environment |
| Iriyama, 2013 | 1.1 Goal setting (behaviour), 1.9 Commitment, 2.3 Self-monitoring behaviour, 3.1 Social support (unspecified), 4.1 Instruction on how to perform a behaviour, 5.1 Information about health consequences, 7.1 Prompts/cues, 10.9 Self-reward, 12.1 Restructuring the physical environment |
| Pechey, 2019 | 12.1 Restructuring the physical environment |
| Roy, 2020 | No BCT codes |
| Sawada, 2015 | No BCT codes |
| Sorensen, 2003 | 1.1 Goal setting (behaviour), 2.3 Self-monitoring of behaviour, 4.1 Instruction on how to perform a behaviour, 6.1 Demonstration of a behaviour |
| van Holland, 2017 | 2.6 Biofeedback |
| van Holland, 2018 | 2.6 Biofeedback |
| van Scheppingen, 2014 | 1.1 Goal setting, 2.3 Self-monitoring of behaviour, 3.1 Social support (unspecified), 4.1 Instruction on how to perform a behaviour, 7.1 Prompts/cues, 12.5 Adding objects to the environment |
| Vasiljevic, 2018 | 7.1 Prompts/cues |
| Vasiljevic, 2019 | 7.1 Prompts/cues |
| Velema, 2018 | 7.1 Prompts/cues, 12.1 Restructuring the physical environment |
| Vitale, 2018 | 4.1 Instruction on how to perform a behaviour, 5.1 Information about health consequences, 7.1 Prompts/cues, 12.1 Restructuring the physical environment |

Abbreviations: BCT – Behaviour Change Technique, BMI – body mass index, LM – lifestyle management, DM – disease management, SBP – systolic blood pressure, ROI – return on investment, WC – waist circumference, WEA – workplace environment audit, NR – not reported

Legend: +ve Significant positive change, -ve Significant negative change, 0 No significant difference
